# An evaluation of indices for quantifying tuberculosis transmission using genotypes of pathogen isolates

**DOI:** 10.1186/1471-2334-6-92

**Published:** 2006-06-07

**Authors:** Mark M Tanaka, Renault Phong, Andrew R Francis

**Affiliations:** 1 School of Biotechnology and Biomolecular Sciences, University of New South Wales, Australia; 2 School of Computing and Mathematics, University of Western Sydney, Australia

## Abstract

**Background:**

Infectious diseases are often studied by characterising the population structure of the pathogen using genetic markers. An unresolved problem is the effective quantification of the extent of transmission using genetic variation data from such pathogen isolates.

**Methods:**

It is important that transmission indices reflect the growth of the infectious population as well as account for the mutation rate of the marker and the effects of sampling. That is, while responding to this growth rate, indices should be unresponsive to the sample size and the mutation rate. We use simulation methods taking into account both the mutation and sampling processes to evaluate indices designed to quantify transmission of tuberculosis.

**Results:**

Previously proposed indices generally perform inadequately according to the above criteria, with the partial exception of the recently proposed Transmission-Mutation Index.

**Conclusion:**

Any transmission index needs to take into account mutation of the marker and the effects of sampling. Simple indices are unlikely to capture the full complexity of the underlying processes.

## Background

The use of molecular markers has provided greater resolution in the characterisation of the spread of infectious disease. In this paper we focus on *M. tuberculosis *because there has been much empirical research effort made to understand its population structure using molecular techniques. Additionally, a number of attempts have been made to develop methods to analyse the resulting data. Although this study is motivated by *M. tuberculosis *data, the results are applicable to all directly transmitted asexual pathogens for which the appropriate molecular data are being generated. In this paper we evaluate a number of indices which have been proposed or used to quantify the extent of an outbreak of tuberculosis. In the case of tuberculosis, where many cases are known to arise from the reactivation of latent pathogen within the host, one approach to quantifying the extent of *recent *transmission uses the proportion of cases in genotype clusters of size two or more. This approach has led to indices in [1,2] which we referred to as *RTI*_*n*-1 _and *RTI*_*n *_in [3]. Another index is an estimate of the number of secondary cases due to each source case. If all cases in a given cluster are assumed to arise from a single source, this number is just the average of the cluster sizes minus one. This approach was introduced in [4] to compare transmissibility between subgroups of the infectious population. We evaluate a modified version of this index to reflect an overall level of secondary transmission (as opposed to that of a subgroup), which we will denote TI˜
 MathType@MTEF@5@5@+=feaafiart1ev1aaatCvAUfKttLearuWrP9MDH5MBPbIqV92AaeXatLxBI9gBaebbnrfifHhDYfgasaacH8akY=wiFfYdH8Gipec8Eeeu0xXdbba9frFj0=OqFfea0dXdd9vqai=hGuQ8kuc9pgc9s8qqaq=dirpe0xb9q8qiLsFr0=vr0=vr0dc8meaabaqaciaacaGaaeqabaqabeGadaaakeaadaaiaaqaaiabdsfaujabdMeajbGaay5adaaaaa@2FBA@. A third approach is to adapt measures of genetic diversity such as Simpson's and Shannon-Weaver's indices. This is again tied to the assumption that the genetic homogeneity of the sample is an indicator of the extent of recent transmission. Finally, a measure of outbreak severity is based on information about the "passage time" or "serial interval" within a host; that is, the average time between the infection of an individual and the transmission to another individual. All other things being equal, the shorter the average passage time, then the faster the outbreak is spreading. Based on this last approach, the Transmission-Mutation Index *TMI *was suggested in [3].

In [3] we argued that it is necessary to account for the rate of mutation of the marker in order to accurately reflect transmission rates using genotypic data. An effective marker-based measure of the level of transmission of an infectious disease

• should be positively related to the growth rate of the infectious population, and

• should not respond either to the mutation rate of the marker, or to the sample size.

Assessing the performance of such indices in relation to the above parameters can be effectively done through simulations in which we are able to control these parameters. In this paper we evaluate a set of five transmission indices using computer simulations. We simulate the process of tuberculosis transmission along with mutation in the marker and random sampling of strains, and determine the effect of various input parameters on the performance of the indices according to the above criteria.

The indices we compare are

• the Recent Transmission Index (*RTI*), an index of clustering derived from those of [1] and [2], which have also been referred to as "the *n *method" and "the *n *- 1 method" [5]

• an index (TI˜
 MathType@MTEF@5@5@+=feaafiart1ev1aaatCvAUfKttLearuWrP9MDH5MBPbIqV92AaeXatLxBI9gBaebbnrfifHhDYfgasaacH8akY=wiFfYdH8Gipec8Eeeu0xXdbba9frFj0=OqFfea0dXdd9vqai=hGuQ8kuc9pgc9s8qqaq=dirpe0xb9q8qiLsFr0=vr0=vr0dc8meaabaqaciaacaGaaeqabaqabeGadaaakeaadaaiaaqaaiabdsfaujabdMeajbGaay5adaaaaa@2FBA@) based on the "Transmission Index" *TI *of [4]

• Simpson's index (as opposed to Simpson's diversity index)

• an index derived from the Shannon-Weaver index (measuring clustering instead of diversity)

• the Transmission-Mutation Index (*TMI*) [3].

For a given data set of genotypes of a pathogen, let *n *be the sample size, *n*_*i *_be the number of isolates of genotype *i *observed in the sample, and *g *be the number of distinct genotypes in the sample. For example, the data in the study [1] can be represented by the list {30^1^, 23^1^, 15^1^, 10^1^, 8^1^, 5^2^, 4^4^, 3^13^, 2^20^, 1^282^} where *i^j ^*means that there are *j *clusters of size *i*. In this case *n = *473 and *g = *326.

The *RTI *is defined to be

RTI=n−gn−1.
 MathType@MTEF@5@5@+=feaafiart1ev1aaatCvAUfKttLearuWrP9MDH5MBPbIqV92AaeXatLxBI9gBaebbnrfifHhDYfgasaacH8akY=wiFfYdH8Gipec8Eeeu0xXdbba9frFj0=OqFfea0dXdd9vqai=hGuQ8kuc9pgc9s8qqaq=dirpe0xb9q8qiLsFr0=vr0=vr0dc8meaabaqaciaacaGaaeqabaqabeGadaaakeaacqWGsbGucqWGubavcqWGjbqscqGH9aqpdaWcaaqaaiabd6gaUjabgkHiTiabdEgaNbqaaiabd6gaUjabgkHiTiabigdaXaaacqGGUaGlaaa@390A@

Note that *RTI* was denoted by RTIn−1*
 MathType@MTEF@5@5@+=feaafiart1ev1aaatCvAUfKttLearuWrP9MDH5MBPbIqV92AaeXatLxBI9gBaebbnrfifHhDYfgasaacH8akY=wiFfYdH8Gipec8Eeeu0xXdbba9frFj0=OqFfea0dXdd9vqai=hGuQ8kuc9pgc9s8qqaq=dirpe0xb9q8qiLsFr0=vr0=vr0dc8meaabaqaciaacaGaaeqabaqabeGadaaakeaacqWGsbGucqWGubavcqWGjbqsdaqhaaWcbaGaemOBa4MaeyOeI0IaeGymaedabaGaeiOkaOcaaaaa@3470@ in [3], and in this form is a minor modification of the more conventional *RTI*_*n*-1 _= (*n - g*)*/n*. We use the former because its maximum value is 1 rather than (*n *- 1)/*n*, and so the maximum is independent of sample size. This modification is simply a scaling by a factor of *n/*(*n *- 1), and hence there is no substantial difference in the behaviours of *RTI *and *RTI*_*n*-1 _(noting that all values of *n *of interest are large).

The TI˜
 MathType@MTEF@5@5@+=feaafiart1ev1aaatCvAUfKttLearuWrP9MDH5MBPbIqV92AaeXatLxBI9gBaebbnrfifHhDYfgasaacH8akY=wiFfYdH8Gipec8Eeeu0xXdbba9frFj0=OqFfea0dXdd9vqai=hGuQ8kuc9pgc9s8qqaq=dirpe0xb9q8qiLsFr0=vr0=vr0dc8meaabaqaciaacaGaaeqabaqabeGadaaakeaadaaiaaqaaiabdsfaujabdMeajbGaay5adaaaaa@2FBA@ is a population level version of the *TI *defined in [4], and is defined to be the average number of secondary infections caused by each source case. Under the assumption that each cluster contains one source case who infects all other members of the cluster, this is equal to ∑_*i*_(*n_i _*- 1)/*g *Hence,

TI˜=n−gg.
 MathType@MTEF@5@5@+=feaafiart1ev1aaatCvAUfKttLearuWrP9MDH5MBPbIqV92AaeXatLxBI9gBaebbnrfifHhDYfgasaacH8akY=wiFfYdH8Gipec8Eeeu0xXdbba9frFj0=OqFfea0dXdd9vqai=hGuQ8kuc9pgc9s8qqaq=dirpe0xb9q8qiLsFr0=vr0=vr0dc8meaabaqaciaacaGaaeqabaqabeGadaaakeaadaaiaaqaaiabdsfaujabdMeajbGaay5adaGaeyypa0ZaaSaaaeaacqWGUbGBcqGHsislcqWGNbWzaeaacqWGNbWzaaGaeiOla4caaa@36B4@

Note that this is also equal to the average cluster size minus one. Incidentally, it has been shown [4] that ReFAST
 MathType@MTEF@5@5@+=feaafiart1ev1aaatCvAUfKttLearuWrP9MDH5MBPbIqV92AaeXatLxBI9gBaebbnrfifHhDYfgasaacH8akY=wiFfYdH8Gipec8Eeeu0xXdbba9frFj0=OqFfea0dXdd9vqai=hGuQ8kuc9pgc9s8qqaq=dirpe0xb9q8qiLsFr0=vr0=vr0dc8meaabaqaciaacaGaaeqabaqabeGadaaakeaacqWGsbGudaqhaaWcbaGaemyzaugabaGaemOrayKaemyqaeKaem4uamLaemivaqfaaaaa@33D9@ = *TI*/(*TI *+ 1) under the assumption that the effective (rapid progression) reproductive number ReFAST
 MathType@MTEF@5@5@+=feaafiart1ev1aaatCvAUfKttLearuWrP9MDH5MBPbIqV92AaeXatLxBI9gBaebbnrfifHhDYfgasaacH8akY=wiFfYdH8Gipec8Eeeu0xXdbba9frFj0=OqFfea0dXdd9vqai=hGuQ8kuc9pgc9s8qqaq=dirpe0xb9q8qiLsFr0=vr0=vr0dc8meaabaqaciaacaGaaeqabaqabeGadaaakeaacqWGsbGudaqhaaWcbaGaemyzaugabaGaemOrayKaemyqaeKaem4uamLaemivaqfaaaaa@33D9@ is less than one. If we use the modified version TI˜
 MathType@MTEF@5@5@+=feaafiart1ev1aaatCvAUfKttLearuWrP9MDH5MBPbIqV92AaeXatLxBI9gBaebbnrfifHhDYfgasaacH8akY=wiFfYdH8Gipec8Eeeu0xXdbba9frFj0=OqFfea0dXdd9vqai=hGuQ8kuc9pgc9s8qqaq=dirpe0xb9q8qiLsFr0=vr0=vr0dc8meaabaqaciaacaGaaeqabaqabeGadaaakeaadaaiaaqaaiabdsfaujabdMeajbGaay5adaaaaa@2FBA@, this gives ReFAST
 MathType@MTEF@5@5@+=feaafiart1ev1aaatCvAUfKttLearuWrP9MDH5MBPbIqV92AaeXatLxBI9gBaebbnrfifHhDYfgasaacH8akY=wiFfYdH8Gipec8Eeeu0xXdbba9frFj0=OqFfea0dXdd9vqai=hGuQ8kuc9pgc9s8qqaq=dirpe0xb9q8qiLsFr0=vr0=vr0dc8meaabaqaciaacaGaaeqabaqabeGadaaakeaacqWGsbGudaqhaaWcbaGaemyzaugabaGaemOrayKaemyqaeKaem4uamLaemivaqfaaaaa@33D9@ = (*n *- *g*)/*n *= *RTI*_*n *- 1_, providing a link between *RTI *and TI˜
 MathType@MTEF@5@5@+=feaafiart1ev1aaatCvAUfKttLearuWrP9MDH5MBPbIqV92AaeXatLxBI9gBaebbnrfifHhDYfgasaacH8akY=wiFfYdH8Gipec8Eeeu0xXdbba9frFj0=OqFfea0dXdd9vqai=hGuQ8kuc9pgc9s8qqaq=dirpe0xb9q8qiLsFr0=vr0=vr0dc8meaabaqaciaacaGaaeqabaqabeGadaaakeaadaaiaaqaaiabdsfaujabdMeajbGaay5adaaaaa@2FBA@. While indices such as *RTI *and TI˜
 MathType@MTEF@5@5@+=feaafiart1ev1aaatCvAUfKttLearuWrP9MDH5MBPbIqV92AaeXatLxBI9gBaebbnrfifHhDYfgasaacH8akY=wiFfYdH8Gipec8Eeeu0xXdbba9frFj0=OqFfea0dXdd9vqai=hGuQ8kuc9pgc9s8qqaq=dirpe0xb9q8qiLsFr0=vr0=vr0dc8meaabaqaciaacaGaaeqabaqabeGadaaakeaadaaiaaqaaiabdsfaujabdMeajbGaay5adaaaaa@2FBA@ are derived using the assumption that the source case is present in the sample, this family of indices can and has been used to study the extent of recent transmission represented by a sample, regardless of whether it contains the source case [6,7].

Two indices based on the ecological indices, Simpson's and an index derived from the Shannon-Weaver index, are defined respectively as

S=∑ni(ni−1)n(n−1)
 MathType@MTEF@5@5@+=feaafiart1ev1aaatCvAUfKttLearuWrP9MDH5MBPbIqV92AaeXatLxBI9gBaebbnrfifHhDYfgasaacH8akY=wiFfYdH8Gipec8Eeeu0xXdbba9frFj0=OqFfea0dXdd9vqai=hGuQ8kuc9pgc9s8qqaq=dirpe0xb9q8qiLsFr0=vr0=vr0dc8meaabaqaciaacaGaaeqabaqabeGadaaakeaacqWGtbWucqGH9aqpdaaeabqaamaalaaabaGaemOBa42aaSbaaSqaaiabdMgaPbqabaGccqGGOaakcqWGUbGBdaWgaaWcbaGaemyAaKgabeaakiabgkHiTiabigdaXiabcMcaPaqaaiabd6gaUjabcIcaOiabd6gaUjabgkHiTiabigdaXiabcMcaPaaaaSqabeqaniabggHiLdaaaa@40D9@

and

CH=∑niln⁡(ni)nln⁡(n).
 MathType@MTEF@5@5@+=feaafiart1ev1aaatCvAUfKttLearuWrP9MDH5MBPbIqV92AaeXatLxBI9gBaebbnrfifHhDYfgasaacH8akY=wiFfYdH8Gipec8Eeeu0xXdbba9frFj0=OqFfea0dXdd9vqai=hGuQ8kuc9pgc9s8qqaq=dirpe0xb9q8qiLsFr0=vr0=vr0dc8meaabaqaciaacaGaaeqabaqabeGadaaakeaacqWGdbWqdaWgaaWcbaGaemisaGeabeaakiabg2da9maaqaeabaWaaSaaaeaacqWGUbGBdaWgaaWcbaGaemyAaKgabeaakiGbcYgaSjabc6gaUjabcIcaOiabd6gaUnaaBaaaleaacqWGPbqAaeqaaOGaeiykaKcabaGaemOBa4MagiiBaWMaeiOBa4MaeiikaGIaemOBa4MaeiykaKcaaaWcbeqab0GaeyyeIuoakiabc6caUaaa@44C8@

See [3] for details. The *TMI *uses a maximum likelihood estimator (MLE) for the compound parameter (*μτ*) to construct an index related to the speed of transmission (*μτ *is the expected number of mutations per case). Namely, if μτ^
 MathType@MTEF@5@5@+=feaafiart1ev1aaatCvAUfKttLearuWrP9MDH5MBPbIqV92AaeXatLxBI9gBaebbnrfifHhDYfgasaacH8akY=wiFfYdH8Gipec8Eeeu0xXdbba9frFj0=OqFfea0dXdd9vqai=hGuQ8kuc9pgc9s8qqaq=dirpe0xb9q8qiLsFr0=vr0=vr0dc8meaabaqaciaacaGaaeqabaqabeGadaaakeaadaqiaaqaaGGaciab=X7aTjab=r8a0bGaayPadaaaaa@30EB@ is the MLE of (*μτ*), where *τ *is the generation time and μ˜
 MathType@MTEF@5@5@+=feaafiart1ev1aaatCvAUfKttLearuWrP9MDH5MBPbIqV92AaeXatLxBI9gBaebbnrfifHhDYfgasaacH8akY=wiFfYdH8Gipec8Eeeu0xXdbba9frFj0=OqFfea0dXdd9vqai=hGuQ8kuc9pgc9s8qqaq=dirpe0xb9q8qiLsFr0=vr0=vr0dc8meaabaqaciaacaGaaeqabaqabeGadaaakeaaiiGacuWF8oqBgaacaaaa@2E78@ is an independent estimate of the mutation rate per unit time *μ *then *TMI *is defined to be μ˜
 MathType@MTEF@5@5@+=feaafiart1ev1aaatCvAUfKttLearuWrP9MDH5MBPbIqV92AaeXatLxBI9gBaebbnrfifHhDYfgasaacH8akY=wiFfYdH8Gipec8Eeeu0xXdbba9frFj0=OqFfea0dXdd9vqai=hGuQ8kuc9pgc9s8qqaq=dirpe0xb9q8qiLsFr0=vr0=vr0dc8meaabaqaciaacaGaaeqabaqabeGadaaakeaaiiGacuWF8oqBgaacaaaa@2E78@/μτ^
 MathType@MTEF@5@5@+=feaafiart1ev1aaatCvAUfKttLearuWrP9MDH5MBPbIqV92AaeXatLxBI9gBaebbnrfifHhDYfgasaacH8akY=wiFfYdH8Gipec8Eeeu0xXdbba9frFj0=OqFfea0dXdd9vqai=hGuQ8kuc9pgc9s8qqaq=dirpe0xb9q8qiLsFr0=vr0=vr0dc8meaabaqaciaacaGaaeqabaqabeGadaaakeaadaqiaaqaaGGaciab=X7aTjab=r8a0bGaayPadaaaaa@30EB@. The *TMI *is intended to be related heuristically to 1/*τ*. In terms of the data-derived parameters *n, g *and *v*_1 _this index is then found to be *TMI *:= μ˜
 MathType@MTEF@5@5@+=feaafiart1ev1aaatCvAUfKttLearuWrP9MDH5MBPbIqV92AaeXatLxBI9gBaebbnrfifHhDYfgasaacH8akY=wiFfYdH8Gipec8Eeeu0xXdbba9frFj0=OqFfea0dXdd9vqai=hGuQ8kuc9pgc9s8qqaq=dirpe0xb9q8qiLsFr0=vr0=vr0dc8meaabaqaciaacaGaaeqabaqabeGadaaakeaaiiGacuWF8oqBgaacaaaa@2E78@(*n *- *g *+ *v*_1_)/*v*_1_, where *v*_1 _is the number of 1-step mutations inferred from the sample data. Given a data set, a more convenient formulation of this is

TMI=μ˜(n−χg−χ)
 MathType@MTEF@5@5@+=feaafiart1ev1aaatCvAUfKttLearuWrP9MDH5MBPbIqV92AaeXatLxBI9gBaebbnrfifHhDYfgasaacH8akY=wiFfYdH8Gipec8Eeeu0xXdbba9frFj0=OqFfea0dXdd9vqai=hGuQ8kuc9pgc9s8qqaq=dirpe0xb9q8qiLsFr0=vr0=vr0dc8meaabaqaciaacaGaaeqabaqabeGadaaakeaacqWGubavcqWGnbqtcqWGjbqscqGH9aqpiiGacuWF8oqBgaacamaabmaabaWaaSaaaeaacqWGUbGBcqGHsislcqWFhpWyaeaacqWGNbWzcqGHsislcqWFhpWyaaaacaGLOaGaayzkaaaaaa@3C80@

where *χ *is the number of connected components in the cluster graph representing the sampled data (under the assumption that each connected component contains exactly one ancestral genotype). Cluster graphs are defined to be graphs constructed from distinct genotypes in a sample, in which edges connect genotypes that may be related by a single mutation event [3].

## Methods

### Evaluating indices

The evaluation of indices involves three stages: simulation of the outbreak and sampling process, computation of indices, and measuring the stability or responsiveness of the indices to the input parameters.

**Figure 1:**
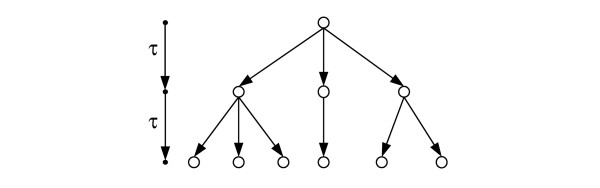
**Example of part of an outbreak across three generations**. The circles and arrows represent infectious cases and transmission events respectively. All transmissions in each generation take place simultaneously; the time between generations is τ.

Consider a discrete time model of outbreaks in which all new infections are produced simultaneously in each generation (or time step). Let *λ *be the average number of new infectious cases produced by a single infectious individual. We shall call this the *average offspring number*. Let *T *be the *generation time *measured in years, that is, the length of the time step between generations (see Figure 1). Equivalently, *t *is the serial interval between successive infections, assumed to be fixed throughout the outbreak across all chains of infection. While a continuous time model involving variable serial intervals might be more realistic it could also involve more parameters. Discrete time models such as the one described above have been used elsewhere to model infectious disease evolution [8]. A good index should at least work in the idealised scenario of this model.

Under the assumption of an infinite susceptible population, the population of infectious individuals *N*_*t *_= *N*(*t*) grows on average according to

*N*_*t *_= *N*_0_*λ *^*t*/*τ*^,     (1)

where *t *is time measured in years, *N*_0 _is the size of the infectious population at time *t *= 0, *τ *is the generation time measured in years, and *λ *is the average offspring number. The value of *t/λ *gives the number of generations since time *t *= 0. In the discrete time model, the function *N*_*t *_is technically only defined when *t/τ *∈ ℕ, since we assume a discrete growth in population. Allowing *t *to vary continuously, however, the growth rate of ln *N*_*t *_is

ddt(ln⁡Nt)=1τln⁡λ.     (2)
 MathType@MTEF@5@5@+=feaafiart1ev1aaatCvAUfKttLearuWrP9MDH5MBPbIqV92AaeXatLxBI9gBaebbnrfifHhDYfgasaacH8akY=wiFfYdH8Gipec8Eeeu0xXdbba9frFj0=OqFfea0dXdd9vqai=hGuQ8kuc9pgc9s8qqaq=dirpe0xb9q8qiLsFr0=vr0=vr0dc8meaabaqaciaacaGaaeqabaqabeGadaaakeaadaWcaaqaaiabdsgaKbqaaiabdsgaKjabdsha0baacqGGOaakcyGGSbaBcqGGUbGBcqWGobGtdaWgaaWcbaGaemiDaqhabeaakiabcMcaPiabg2da9maalaaabaGaeGymaedabaacciGae8hXdqhaaiGbcYgaSjabc6gaUjab=T7aSjabc6caUiaaxMaacaWLjaWaaeWaaeaacqaIYaGmaiaawIcacaGLPaaaaaa@44FD@

Since ln *N*_*t *_grows monotonically with *N*_*t*_, this growth rate gives an indication of the speed with which an outbreak is spreading. Note that this rate is independent of *N*_*t*_, the mutation rate *μ *and the proportion of cases sampled *φ*. These considerations imply that an index reflecting the growth rate of the outbreak should show

1. a positive relationship with the average offspring number (*λ*) and the reciprocal of the generation time (1/*τ*), and

2. low sensitivity to

(a) the mutation rate *μ *per unit time,

(b) the sampling proportion *φ*, and

(c) the size of the infectious population *N*_*t*._

Mixed relationships with *λ *and 1/*τ *might still be satisfactory provided the relationship with 1τ
 MathType@MTEF@5@5@+=feaafiart1ev1aaatCvAUfKttLearuWrP9MDH5MBPbIqV92AaeXatLxBI9gBaebbnrfifHhDYfgasaacH8akY=wiFfYdH8Gipec8Eeeu0xXdbba9frFj0=OqFfea0dXdd9vqai=hGuQ8kuc9pgc9s8qqaq=dirpe0xb9q8qiLsFr0=vr0=vr0dc8meaabaqaciaacaGaaeqabaqabeGadaaakeaadaWcaaqaaiabigdaXaqaaGGaciab=r8a0baaaaa@2F78@ ln *λ *is correct.

It should be expected that all indices would be sensitive to the proportion of cases sampled *φ *when this proportion is low, but it would be desirable for an index to become insensitive to *φ *beyond a certain value. Furthermore, since in general the genetic configuration of the set of founders (see below) is unknown, a good index should satisfy the above criteria regardless of this configuration.

A basic outline of our methodology is as follows.

1. For a given set of parameters representing mutation rate *μ *average offspring number *λ*, generation time *τ*, sampling proportion *φ*, the number *N *of infectious cases in the population over the sampling period, and a set of founders **f **(of which more is explained below)

(a) simulate an outbreak of infectious disease using the input parameters *λ*, *μ*, and *τ*, stopping when the sampling period ends,

(b) for each generation in which sampling occurs, take a sample of size *n *= *φN *cases, and

(c) compute the various indices from the overall sample.

2. Vary one of the parameters and repeat.

3. Graph indices against each parameter to assess index responses.

### Simulations of outbreaks

We simulate the spread of an outbreak stochastically by assuming the outbreak takes the form of a modified Galton-Watson branching process in which mutation of the pathogen at the marker locus may occur at any transmission with a given probability *μ*_*p *_(see below). In such a process, an outbreak is represented by a set of rooted transmission trees (as defined in [3], see also Figure 1 for a small example) whose roots are the set of founders **f**. By "founders" we mean a set of infectious hosts, potentially involving different genotypes, in the population at time *t *= 0. In the transmission trees (the "forest") arising from these simulations, each vertex (or "node") represents an infectious individual and each edge a transmission event. Thus the set of infectious individuals at time *t *is the set of leaves (terminal vertices) of the transmission forest at generation *t/τ*.

The fundamental input parameter of the branching process is the average offspring number *λ*, which is assumed to be constant for the duration of the outbreak. That is, we specify a value for *λ *together with a distribution of the numbers of offspring of individuals at any time. The distribution of the offspring number (the number of new infections per individual) is assumed to be Poisson with parameter *λ*. All transmissions from a given generation to the next are completed before transmissions from the next generation begin. The simulation is stopped after the sampling period (set at two years) ends.

We assume the genotypes of the founding clusters are "sufficiently distinct", so that the only way two genotypes can be a single mutation step apart is if they have arisen from the same founder. We make the infinite alleles assumption, so that each mutation event is assumed to produce a previously non-existent allele.

The mutation probability *μ*_*p *_is obtained from the rate *μ *by modelling mutation as a time-homogeneous stochastic process [9] using

*μ*_*p *_= 1 - *e*^-*μτ *^    (3)

where *τ *is the generation time, fixed for each outbreak.

Sampling takes place from those generations that occur during the last two years of the outbreak. The number of generations occurring within the last two years of the outbreak is *k *= ⌋2/*τ*⌌ (where ⌋*x*⌌ is the greatest integer less than or equal to *x*). In this period, the total number of infectious individuals is on average *N*_*b *_+ *λN*_*b *_+ .. + *λ*^*k*^*N*_*b*_, where *N*_*b*_ is the population size at the first generation in which sampling takes place. Setting this quantity to be *N *(the number of infectious cases over the sampling period) we have *N*_*b *_= *N*/(1 + *λ *+ .. + *λ*^*k *^). In the simulation, we therefore commence sampling in the first generation at which *N*_*b *_is exceeded. Samples are constructed from the infectious population by choosing a proportion *φ *of individuals randomly without replacement within each generation of the sampling period. Note that fixing of *N *as an input parameter models an epidemiological response to an outbreak, which is more realistic than fixing the number of generations in the simulation.

The parameters of the model (apart from the sizes of the clusters of the founders) are summarised in Table 1 along with the standard set of values used in the simulation. The parameter ranges in this study are chosen to be consistent with values in the tuberculosis literature. For instance, the default value of *τ *is drawn from an estimate of serial intervals in [10] and the default value of *μ *is based on [9]. The value of *λ *is based on estimates of the basic and net reproductive number in [11,12]. The sensitivity of indices to the proportion of cases sampled is important, and hence *φ *= *n/N *is one of the parameters we allow to vary. The values of the parameters are varied one at a time. Although the present study covers a wide variation of parameters, it cannot cover all points in the full parameter space. An open question for each of the indices is to determine the regions of this space in which accurate inferences can be drawn.

**Table 1:** Parameters and their values. This table gives a description of the parameters and the default values they are given in the simulations.

Parameter	Description	Default value
*λ*	Average offspring number	2
*μ*	Mutation rate of the marker in use	0.2 per year
*τ*	Generation time	1 year
*φ*	Sampling proportion	0.6
*N*	Outbreak size	1000

**Figure 2:**
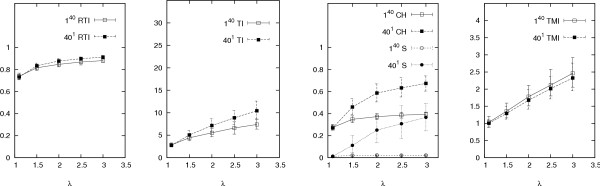
**Responses of indices over *λ***. Other parameters are as in Table 1 (*φ *= 0.6, *τ *= 1 year, *μ *= 0.2 per year and *N *= 1000). The different founding sets are represented by 40^1 ^and 1^40^, meaning one cluster of size forty and forty clusters of size one respectively. The error bars indicate the central 90% of simulated values.

**Figure 3:**
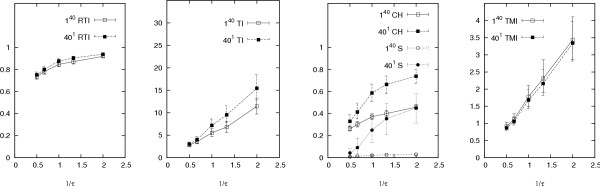
**Responses of indices over 1/*τ***. Other parameters are as in Table 1 (*φ *= 0.6, *λ *= 2, *μ *= 0.2 per year and *N *= 1000). The different founding sets are represented by 40^1 ^and 1^40^, meaning one cluster of size forty and forty clusters of size one respectively. The error bars indicate the central 90% of simulated values.

**Figure 4:**
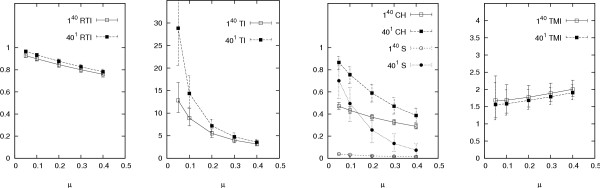
**Responses of indices over ***μ*. Other parameters are as in Table 1 (*φ *= 0.6, *λ *= 2, *τ *= 1 year and *N *= 1000). The different founding sets are represented by 40^1 ^and 1^40^, meaning one cluster of size forty and forty clusters of size one respectively. The error bars indicate the central 90% of simulated values. The upper value of the bar for TI˜
 MathType@MTEF@5@5@+=feaafiart1ev1aaatCvAUfKttLearuWrP9MDH5MBPbIqV92AaeXatLxBI9gBaebbnrfifHhDYfgasaacH8akY=wiFfYdH8Gipec8Eeeu0xXdbba9frFj0=OqFfea0dXdd9vqai=hGuQ8kuc9pgc9s8qqaq=dirpe0xb9q8qiLsFr0=vr0=vr0dc8meaabaqaciaacaGaaeqabaqabeGadaaakeaadaaiaaqaaiabdsfaujabdMeajbGaay5adaaaaa@2FBA@ at *μ *= 0.05 (out of the range of the graph) is 40.3.

**Figure 5:**
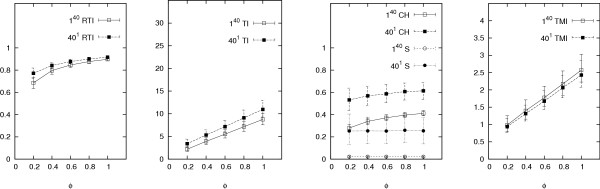
**Responses of indices over *φ***. Other parameters are as in Table 1 (*τ *= 1 year, *μ *= 0.2 per year, *λ *= 2 and *N *= 1000). The different founding sets are represented by 40^1 ^and 1^40^, meaning one cluster of size forty and forty clusters of size one respectively. The error bars indicate the central 90% of simulated values.

**Figure 6:**
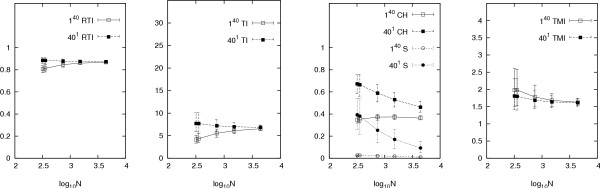
**Responses of indices over log_10_(*N*)**. Other parameters are as in Table 1 (*φ *= 0.6, *λ *= 2, *τ *= 1 year and *μ *= 0.2 per year). The different founding sets are represented by 40^1 ^and 1^40^, meaning one cluster of size forty and forty clusters of size one respectively. The error bars indicate the central 90% of simulated values.

Our results are presented in Figures 2, 3, 4, 5, 6, which graph the behaviour of the indices against average offspring number *λ*, the reciprocal of the generation time *τ*, mutation rate *μ*, sampling proportion *φ *and the logarithm of the infectious population size over the sampling period *N*, respectively. Each graph shows the results of two sets of simulations, corresponding to the different founding sets 40^1 ^and 1^40^, representing one cluster of size forty and forty clusters of size one respectively (other configurations, including some from actual data sets, produced similar results (not shown) to these two extremes). Each point on each graph represents index values averaged over one thousand runs of the simulation. The error bars indicate the central 90% of index values (among the thousand runs). Simulations were also run with the distribution of the offspring number being geometric instead of Poisson, however these gave similar results (results not shown).

## Results and discussion

In both of its published standard forms (as the *n*-method and the *n *- 1-method), the *RTI *has been assessed theoretically as a measure of actual clusteredness in the infectious population, and has been found to systematically underestimate the real clusteredness when computed on the basis of a sample [5,13-15]. Although the same question could be asked about other indices, the present study seeks to answer a different question: whether an index provides a meaningful point of comparison between different outbreaks, regardless of how accurately the index value from a sample corresponds to the actual population value. We do this for each of the indices described above by assessing their responses to changes in the parameters of the outbreak.

Recall that it is desirable for indices to respond positively to mean offspring number and the reciprocal of the generation time, while being stable with respect to mutation rate, sample size and outbreak size (see the Methods section).

The indices generally respond well to 1/*τ *and *λ *(Figures 2 and 3). As *λ *decreases, fixing the infectious population size parameter *N *forces an increase in the number of generations. The increased number of generations allows more time for mutation events and therefore leads to a more genetically heterogeneous data set – that is, one that contains many different genotypes. This then explains the appropriate responses of the indices measuring clustering to *λ*. On the other hand, as the generation time *τ *is decreased (increasing 1/*τ*) and *μ *is kept constant, *μ*_*p *_decreases according to Equation (3). This leads to a more homogeneous outbreak, which similarly explains why the indices respond well to 1/*τ*.

In contrast, as discussed in [3], increasing *μ *leads to a more heterogeneous outbreak and so the indices that measure clustering without accounting for mutation decline in response (Figure 4). Because these indices are only intended to reflect the clusteredness of a sample, they cannot allow comparisons over varying mutation rates (or different markers). The *TMI is *consequently the only index here that does not decline with *μ*. In fact, it increases very slightly, suggesting that the correction for mutation in the *TMI *is not perfect.

All indices except *S *increase with the sampling proportion *φ *(Figure 5). In the case of the *RTI*, this response has already been reported [5,13-15]. This sensitivity to *φ *is because as the sampling proportion increases, the probability of selecting a genotype already in the sample increases, leading to greater clusteredness in the sample. While TI˜
 MathType@MTEF@5@5@+=feaafiart1ev1aaatCvAUfKttLearuWrP9MDH5MBPbIqV92AaeXatLxBI9gBaebbnrfifHhDYfgasaacH8akY=wiFfYdH8Gipec8Eeeu0xXdbba9frFj0=OqFfea0dXdd9vqai=hGuQ8kuc9pgc9s8qqaq=dirpe0xb9q8qiLsFr0=vr0=vr0dc8meaabaqaciaacaGaaeqabaqabeGadaaakeaadaaiaaqaaiabdsfaujabdMeajbGaay5adaaaaa@2FBA@ and *TMI *respond almost linearly to *φ*, *RTI *and *C*_*H *_respond far better, by beginning to level off as the sample reaches about half the total population. Although *S *is completely unresponsive to *φ*, it also carries no information at all when the founding set is 1^40^.

The responses of the indices to infectious population size *N *are mild, except for *C*_*H *_and *S *(Figure 6). A common feature is that the value of each index for different founding sets appears to converge as *N *increases. This suggests that with the extra time provided by increasing *N*, the configuration of genotypes in the data becomes less dependent on the founding configuration in the population. Generally, however, the indices are not strongly dependent on *N*. Note that because *φ *is fixed while *N *varies for this figure, the sample size *n *increases with *N*.

The set of founders has a mild effect on indices measuring clustering (*RTI*, TI˜
 MathType@MTEF@5@5@+=feaafiart1ev1aaatCvAUfKttLearuWrP9MDH5MBPbIqV92AaeXatLxBI9gBaebbnrfifHhDYfgasaacH8akY=wiFfYdH8Gipec8Eeeu0xXdbba9frFj0=OqFfea0dXdd9vqai=hGuQ8kuc9pgc9s8qqaq=dirpe0xb9q8qiLsFr0=vr0=vr0dc8meaabaqaciaacaGaaeqabaqabeGadaaakeaadaaiaaqaaiabdsfaujabdMeajbGaay5adaaaaa@2FBA@, *C*_*H *_and *S*), with the diverse founding set 1^40 ^resulting in lower index values. This is the result of the increased diversity forced by this configuration. The *TMI *and the *RTI *appear relatively insensitive to the configuration of founders within this region of the parameter space.

In summary, the clustering indices *RTI*, TI˜
 MathType@MTEF@5@5@+=feaafiart1ev1aaatCvAUfKttLearuWrP9MDH5MBPbIqV92AaeXatLxBI9gBaebbnrfifHhDYfgasaacH8akY=wiFfYdH8Gipec8Eeeu0xXdbba9frFj0=OqFfea0dXdd9vqai=hGuQ8kuc9pgc9s8qqaq=dirpe0xb9q8qiLsFr0=vr0=vr0dc8meaabaqaciaacaGaaeqabaqabeGadaaakeaadaaiaaqaaiabdsfaujabdMeajbGaay5adaaaaa@2FBA@, *C*_*H *_and *S *respond appropriately to *λ *and 1/*τ*, but poorly to *μ *and *φ *(with the exception of *S *in relation to *φ*). The *TMI *responds well to both *λ *and 1/*τ*. It is more stable with respect to the mutation rate, as intended, and is less sensitive to the founding set. Unfortunately, this index is rather sensitive to sampling proportion *φ*.

## Conclusion

Indices measuring the extent of transmission can be evaluated by examining their responsiveness to key parameters, including average offspring number *λ*, generation time *τ*, mutation rate *μ*, sampling proportion *φ *and infectious population size *N*. Namely, a good index should respond positively to *λ *and 1/*τ*, and should not respond to *μ*, *φ *and *N*.

We have shown using computer simulations that five indices for measuring the extent of ongoing transmission generally do not perform well according to our criteria. The Transmission-Mutation Index *TMI *is promising in that a high *TMI *would be more likely due to high 1/*τ *than high *μ*, whereas the other indices respond too strongly to *μ *to allow such an inference. We caution, however, that the *TMI *requires an independent estimate of the mutation rate, which adds a source of uncertainty. Note that this uncertainty is not accounted for in the simulation as values for the mutation rate are set *a priori*. Furthermore, the *TMI *appears to be too sensitive to *φ *to permit reliable inferences.

Theoretical studies such as [3,5,13] and the present paper have begun to illuminate aspects of a range of summary statistics. These findings make it difficult to anticipate complete success from approaches that summarise data through simple indices. The main reason for this is simply that they cannot capture the complexities of the underlying processes, and so these approaches may intrinsically lead to error in inferences. The need remains to develop methods to draw quantitative inferences from the genotypes sampled from an outbreak. The weaknesses that have been exposed should stimulate further enquiry into approaches involving more realistic models and explicit statistical procedures.

## Competing interests

The author(s) declare that they have no competing interests.

## Authors' contributions

MMT and ARF conceived the project and the methodology, and wrote the manuscript. RP wrote the code, ran the simulations and helped draft the manuscript. All authors have read and approved the manuscript.

## Pre-publication history

The pre-publication history for this paper can be accessed here:



## References

[B1] Small PM, Hopewell PC, Singh SP, Paz A, Parsonnet J, Ruston DC, Schecter GF, Daley CL, Schoolnik GK (1994). The epidemiology of tuberculosis in San Francisco: A population-based study using conventional and molecular methods. N Engl J Med.

[B2] Alland D, Kalkut G, Moss A, McAdam R, Hahn J, Bosworth W, Drucker E, Bloom B (1994). Transmission of tuberculosis in New York City. An analysis by DNA fingerprinting and conventional epidemiologic methods. N Engl J Med.

[B3] Tanaka MM, Francis AR (2005). Methods of quantifying and visualising outbreaks of tuberculosis using genotypic information. Infect Genet Evol.

[B4] Borgdorff MW, Nagelkerke N, van Soolingen D, de Haas PE, Veen J, van Embden JD (1998). Analysis of tuberculosis transmission between nationalities in the Netherlands in the period 1993–1995 using DNA fingerprinting. Am J Epidemiol.

[B5] Glynn JR, Vynnycky E, Fine PE (1999). Influence of sampling on estimates of clustering and recent transmission of *Mycobacterium tuberculosis *derived from DNA fingerprinting techniques. Am J Epidemiol.

[B6] Kempf MC, Dunlap NE, Lok KH, Benjamin WHJ, Keenan NB, Kimerling ME (2005). Long-term molecular analysis of tuberculosis strains in Alabama, a state characterized by a largely indigenous, low-risk population. J Clin Microbiol.

[B7] Glynn JR, Crampin AC, Yates MD, Traore H, Mwaungulu FD, Ngwira BM, Ndlovu R, Drobniewski F, Fine PEM (2005). The importance of recent infection with *Mycobacterium tuberculosis *in an area with high HIV prevalence: a long-term molecular epidemiological study in Northern Malawi. J Infect Dis.

[B8] Antia R, Regoes RR, Koella JC, Bergstrom CT (2003). The role of evolution in the emergence of infectious diseases. Nature.

[B9] Rosenberg NA, Tsolaki AG, Tanaka MM (2003). Estimating change rates of genetic markers using serial samples: applications to the transposon IS*6110 *in *Mycobacterium tuberculosis*. Theor Popul Biol.

[B10] ten Asbroek AH, Borgdorff MW, Nagelkerke NJ, Sebek MM, Deville W, van Embden JD, van Soolingen D (1999). Estimation of serial interval and incubation period of tuberculosis using DNA fingerprinting. Int J Tuberc Lung Dis.

[B11] Blower SM, McLean AR, Porco TC, Small PM, Hopewell PC, Sanchez MA, Moss AR (1995). The intrinsic transmission dynamics of tuberculosis epidemics. Nature Med.

[B12] Vynnycky E, Fine PE (1998). The long-term dynamics of tuberculosis and other diseases with long serial intervals: implications of and for changing reproduction numbers. Epidemiol Infect.

[B13] Murray M (2002). Sampling bias in the molecular epidemiology of tuberculosis. Emerg Infect Dis.

[B14] Murray M (2002). Determinants of cluster distribution in the molecular epidemiology of tuberculosis. Proc Natl Acad Sci USA.

[B15] Murray M, Alland D (2002). Methodological problems in the molecular epidemiology of tuberculosis. Am J Epidemiol.

